# Perceived legitimacy of normative expectations motivates compliance with social norms when nobody is watching

**DOI:** 10.3389/fpsyg.2015.01413

**Published:** 2015-10-06

**Authors:** Giulia Andrighetto, Daniela Grieco, Luca Tummolini

**Affiliations:** ^1^Institute of Cognitive Sciences and Technologies, Italian National Research CouncilRome, Italy; ^2^Robert Schuman Center for Advanced Studies, European University InstituteFiesole, Italy; ^3^Economics Department, Bocconi UniversityMilan, Italy

**Keywords:** social norm compliance, empirical and normative expectations, trust, guilt aversion, verbal communication, resentment hypothesis, legitimacy

## Abstract

Three main motivations can explain compliance with social norms: fear of peer punishment, the desire for others' esteem and the desire to meet others' expectations. Though all play a role, only the desire to meet others' expectations can sustain compliance when neither public nor private monitoring is possible. Theoretical models have shown that such desire can indeed sustain social norms, but empirical evidence is lacking. Moreover it is unclear whether this desire ranges over others' “empirical” or “normative” expectations. We propose a new experimental design to isolate this motivation and to investigate what kind of expectations people are inclined to meet. Results indicate that, when nobody can assign either material or immaterial sanctions, the perceived legitimacy of others' normative expectations can motivate a significant number of people to comply with costly social norms.

## Introduction

Our everyday life is structured around many recurrent social situations in which we choose to act in ways that conform at least to one underlying social norm. Greeting is just one mundane example in which we typically behave as we are expected to: one is prepared to extend the right hand to greet, but to bow instead if bowing is expected (as it is in Japan, for instance). Queuing for a taxi at the airport is another common one: if people are expected to wait in line before claiming a free taxi, they typically conform to this shared expectation. When no such shared expectation is present, people behave in much less predictable ways. Why do people typically comply with social norms?

One standard explanation for social situations resembling the greeting example is that the decision to comply stems from the common interest to coordinate over some compatible behaviors. Here the problem is that there might be more than one way in which we could coordinate: to express our mutual respect, we could both shake our right hands or we could both bow. In these situations, expecting others to behave in a definite manner, and being likewise expected, would give one a sufficient reason to conform to the shared expectation simply out of our independent (and compatible) self-interests. In other words, these social situations are regulated by that special kind of social norms that are *conventions* (Lewis, 1969; Sugden, 2004; Bicchieri, 2006; Tummolini et al., 2013).

This explanation, however, does not easily extend also to our second example. Indeed when queuing for a taxi, we are not taking part to a coordination game but to a *mixed-motive* one: a situation in which our interests are not perfectly aligned. Each of us, in fact, would like to take a taxi as fast as possible, and thus would benefit more by jumping the line—if he or she expects all the others to stand there quietly—than by joining in with the rest and waste time. So, even if the next guy is expected to fall in line, he could do better by going straight to the taxi: if so, why, typically, will he stand there with the others? In other words, why should a person be motivated to comply with social norms, when this is contrary to self-interest?

The most common answer is that the violator will be punished by those following the norm (for a review see Fehr and Fischbacher, 2004a). The fear of being punished by peers is certainly an important motive behind social norm compliance and the role of punishment in the evolution and maintenance of social norms is well understood (Boyd et al., 2003, 2010). Indeed, even if queuing is not formally enforced, the majority of those standing in line are frustrated by the occasional jumper, and some of them may also be prepared to actively intervene to inflict material costs on the violator, thereby making the decision to comply actually aligned with his or her self-interest (Fehr and Gächter, 2002; Fehr and Fischbacher, 2004b).

Punishment has received much attention in the literature on social norms, despite the fact that, outside the lab, the imposition of material costs on norm violators by peers is more an exception than the rule (Guala, 2012).

In fact, people might be more readily prepared to sanction one another's behavior with more immaterial currencies, by, for instance, rewarding those who behave as expected, with *esteem* or *respect* and by withdrawing such immaterial benefits from those who violate (Brennan and Pettit, 2000). In a seminal field experiment on intrusions in queues (Milgram et al., 1986), for instance, only 10% of reactions were carried out with some form of material punishment, while the rest employed either verbal or nonverbal expressions of disapproval. These reactions to violations can actually be effective if people care about their *status* vis-à-vis one another, and give some weight to the public perception of their behavior. This additional weight may explain conformity even without facing the prospect of material punishment (see for instance Bernheim, 1994)^1^. Differently from fear of punishment, the desire for status or esteem requires that people also value one another's opinions, that is, they should value also what others *think* of them as opposed to care only for what others might *do* to them. Moreover, in this view, such an appetite for esteem (and aversion to the risk of losing it) would be connected to more complex emotions than mere fear. Shame, for instance, is intrinsically related to one's self-esteem and the esteem that others grant us, and presupposes a desire to be positively evaluated by others relative to a shared standard, which in this case is provided by the social norm itself (Miceli and Castelfranchi, 1998).

Still, in many situations, the violator cannot be easily spotted, if not else because nobody is watching all the time. If fear of peer punishment or the desire for others' esteem (and to avoid shame) were the sole motivations behind social norm compliance, everyone would be prepared to violate when sure to be unseen. Consider, for instance, a smoker who lives in a country where cigarette butts are routinely thrown on the ground. When traveling to a place where he knows that he is expected to throw them in the bin, the smoker would not be careless because he would expect that someone might physically step in. He might be less concerned with the withdrawal of esteem by these strangers, though, since he might feel that he has not much in common with them. Were nobody there to police, however, there would be nobody to impose costs or to negatively assess, and thus the smoker could as well ignore the material or immaterial consequences for the occasional violation. Still, we suggest, he might feel the pressure of being expected to conform anyway, and this pressure could be enough to comply.

To explain social norm compliance when there is no private or public monitoring, or, more in particular, when there is no scope for punishment—as in one-shot encounters between strangers—or for the assignment or withdrawal of esteem by relevant peers, an additional motivation should then be considered.

As far as social norm compliance is concerned, the desire to avoid punishment and the desire for others' esteem have something in common: both motivate to behave as expected for instrumental reasons only. Hence, a natural alternative is that people might be *intrinsically* motivated to comply with social norms, which would be the case, for instance, if they desired to meet others' expectations *per se*. How could such a motivation be understood?

In the context of social norms, a desire of this kind has been proposed by Robert Sugden with his *resentment hypothesis* (Sugden, 2000, 2004). In particular, in Sugden's view, when a social norm exists, people come to have *normative expectations* about each other, which are considered as nothing but “a special kind of empirical expectation which one person holds about another person's action” and which have “some power to motivate that other person to act in conformity with it” (2000, p. 115). Consider, for instance, the social norm prevailing in US according to which diners typically leave a 15% tip. If I am traveling there and I have been told about this custom, I will know that the waiter expects that I leave a 15% tip as well: failing to live up to such expectation would plausibly have some psychological cost for me^2^. Intuitively, once the waiter reasonably expects customers to leave a 15% tip, he would be disappointed at frustration of his expectations and would feel resentment toward those who frustrate them. According to Sugden's Resentment hypothesis, awareness of this tendency induces an aversion toward doing any action that would trigger it^3^. In other words, people are intrinsically averse to be the target of others' resentment. Such an aversion is displayed, for instance, by someone who wants to avoid the *guilt* he would experience if he were to harm others relative to what they *expected* to obtain (Baumeister et al., 1994; Battigalli and Dufwenberg, 2007). Inspired by this view, Charness and Dufwenberg (2006) have proposed that *guilt aversion* might provide a micro-foundation for social norm compliance. Referring to the same tipping example, they suggest that once “there is a norm, it shapes the [waiter]'s expectation, and the customer lives up to this expectation because he would feel guilty if he did not” (Charness and Dufwenberg, 2006, p. 1596).

Both Sugden's resentment hypothesis and Charness and Dufwenberg's guilt aversion theory point to a motivation to behave as expected that implies intrinsic compliance with social norms. This motivation differs from fear of punishment and from the desire for others' esteem since these motives provide only instrumental reasons for compliance. Both proposals, however, also reduce *normative* expectations to *empirical* ones. That is, in both theories, whether one has an empirical justified belief that someone else will act in a certain way (empirical expectation) or a normatively justified belief about how someone *ought* to behave (normative expectation) is irrelevant to explain the motivating power of these expectations. This implies, unfortunately, that the desire to meet others' *empirical* expectations cannot but be a form of altruism after all. If one gives some weight in one's own decision making on whether some stranger, as a consequence of one's violation, achieves *less* than what he or she *expected* to, then this belief-dependent motivation is just another kind of *social preference* for the stranger's welfare (Attanasi and Nagel, 2008).

There is, however, also another way to understand this motivation to behave as expected.

Cristina Bicchieri, in her seminal theory of social norms (Bicchieri, 2006), has, for instance, suggested that an under-explored motive for social norm compliance is the *perceived legitimacy* of others' *normative* expectations (Bicchieri, 2006, p. 24). In her view, normative expectations are understood as what one believes that others think one *ought* to do, i.e., what one is normatively expected to do^4^. Together with the fact that a given social norm is indeed regularly followed and with appropriate empirical expectations (i.e., the first-order belief that others will comply with the social norm), normative expectations provide a reason to follow the social norm. Aside from the motivations discussed above (fear of punishment and the desire for esteem), an important reason is that one perceives as *legitimate* what one is normatively expected to do. Perceived legitimacy of normative expectations reflects the fact that a social norm is accepted and that one acknowledges the others' normative claim for expecting a certain action^5^. Being prepared to offer an excuse for one's behavior is, for instance, a signal that one perceives the normative claim of another one as legitimate. Being willing to accept punishment is another (Faillo et al., 2013). Without aiming to unpack perceived legitimacy any further, it is here important to emphasize that *perceived legitimacy pertains to normative expectations and not to empirical ones*^6^: to the motivational power of beliefs about *what others think that one “ought” to do*. We hypothesize that perceived legitimacy may be sufficient to motivate some people to behave as expected, and that, if it were so, these people would be motivated to live up to the expectations of others *irrespective of being watched*.

Thus, in theory, both the desire to meet the *empirical* expectations of others (the aversion to be the target of others' resentment or to feel the guilt for having disappointed them^7^) and the desire to meet their *normative* ones (perceived legitimacy) predict that compliance with a social norm is possible even if nobody is watching. Which of the two is the most relevant in practice is, however, an empirical question.

The aim of this paper is then to present a new experimental design to isolate the desire to meet others' expectations from other possible motivations to comply with social norms like fear of punishment and the desire for others' esteem. Moreover, our aim is to establish whether this desire ranges over others' *empirical* or *normative* expectations.

## Disentangling the motivations for social norm compliance: three requirements

Disentangling in an experiment the three main motivations behind social norm compliance that we have identified—fear of peer punishment, the desire for others' esteem (and to avoid shame), the desire to meet others' expectations—is not an easy task.

While punishment can be easily controlled for by not giving such an option to experimental subjects and by avoiding repeated encounters (i.e., by using one-shot games with anonymous subjects who cannot punish one another), isolating the desire for others' esteem from the desire to meet others' expectations is more challenging.

The reason for this methodological difficulty is that both motivations are easily confounded since both depend on information about beliefs of others.

From a theoretical point of view, this information is, however, different.

The desire for others' esteem depends on what one believes that another person believes about oneself *at the end of an interaction* (i.e., on *ex-post* information about beliefs of others; see Tadelis, 2011). For instance, if I am worried to lose my status with another one, I can decide to act in accordance with a social norm in order to avoid that another one will believe that I am a “bad” guy after our interaction is completed. This negative assessment would diminish my status, and, if I anticipate it, I may decide to conform to the social norm (Bernheim, 1994).

On the contrary, the desire to meet others' expectations depends on what one believes that others believe about oneself *before the interaction takes place* (i.e., on *ex-ante* information about beliefs of others). This means that, if I have such desire, I am concerned with not disappointing others' already existing expectations about me, something that would happen if I violated the social norm. Thus, to avoid this, I may choose to conform (Sugden, 2000, 2004; Bicchieri, 2006).

As a consequence, the first requirement to disentangle these two motivations experimentally is to be able to *manipulate subjects' accessibility to both* ex-post *and* ex-ante *information about beliefs of others*.

From a formal point of view, the best tool to represent these motivations is provided by *psychological game theory* (Geanakoplos et al., 1989). Psychological game theory, PGT, is a generalization of traditional game theory in which an individual's utility does not only depend on which actions are chosen by the parties but also on their beliefs, and thus it can be used to model belief-dependent preferences of the kind we are interested in. Moreover, with *dynamic* psychological game theory (Battigalli and Dufwenberg, 2009), one is also able to capture the role of higher-order beliefs, of beliefs of others, and also their update during interaction, which is exactly what understanding the desire for esteem or the desire to meet others' expectations require. For these reasons, leading contributions in the theory of social norms have adopted precisely this framework (e.g., Bernheim, 1994; Sugden, 2000, 2004; Bicchieri and Sontuoso, 2015).

From an experimental point of view, however, being able to control the information that subjects can access and the way they form and update their beliefs is more difficult. Notwithstanding how much anonymity is protected and how difficult it is to infer something about others, subjects may still take into account what others might think of them.

One way out of this methodological complexity is to create a design in which *subjects, who are mainly driven by one motivation will choose differently from those driven by another one*. Our second requirement is then to have a design matching this feature.

Finally, since we are concerned with social norm compliance, there should be a reliable way to make a social norm sufficiently *salient* for subjects (Cialdini et al., 1991; Bicchieri, 2006; Conte et al., 2014), since if nothing points to a social norm, it is unclear how subjects might decide to conform to it. A typical way in which a social norm can be made salient is if subjects are allowed to engage in *pre-play verbal communication* by sending, for instance, a written message. According to Bicchieri (2002), for instance, verbal communication typically inflates cooperation rates in social dilemma kind of situations (for reviews see Sally, 1995 and Balliet, 2010) precisely because it has the effect of making specific behavioral rules situationally salient for participants, and this effect induces the individuals to orient their attention to the strategies prescribed by such rules (see also Andrighetto et al., 2013)^8^. Thus, our third and last requirement is that of *using verbal communication to make a social norm salient between the subjects*.

## Experimental design and procedures

### A risky trust game with communication, exposure and a “wiggle room”

Charness and Dufwenberg (2006; C&D henceforth) have proposed an elegant design to test whether the effect of verbal communication on cooperation—trust and trustworthiness, in particular—can be explained by *guilt aversion*, an important way to model the desire to meet others' expectations (for the theoretical model see Battigalli and Dufwenberg, 2007). Given the role that psychological game theory plays in their experiment and the use of communication, this design offers an ideal starting point for our aims.

**Figure 1:**
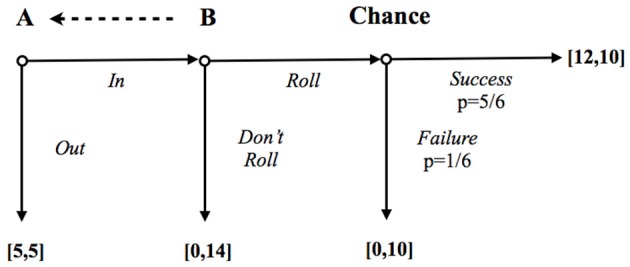
**The risky Trust game of Charness and Dufwenberg (2006)**. The dashed arrow means that the trustee (B) can unilaterally send a message to the trustor (A). Payoffs are expressed in Euros.

C&D design is a trust game with a hidden action, a risky component, and with the possibility for the trustee to send a non-binding message (see Figure 1).

In this variant of the Trust game, a trustor (A) is endowed with a certain amount of money and can choose a safe option (5€, 5€), thereby deciding not to enter in the game (OUT option), or to transfer the endowment (IN option) to the matched trustee (B). By choosing the IN option, the amount of money transferred to B is multiplied. Before making this decision, each B has the possibility to send a non-binding message to his matched A. After having decided whether or not to send the message, B chooses whether to ROLL or DON'T ROLL a six-sided dice. If B decides not to roll the dice, the amount of money remains with B (0€, 14€); otherwise, by rolling the dice, there is 1/6 probability that A will receive 0 and B 10€ and 5/6 probability that A will receive 12€ and B 10€. Crucially, in the original C&D design, trustors could not directly observe the actions of their counterparts, and thus could not discriminate a bad outcome due to untrustworthy behavior from mere bad luck^9^.

The hidden action component and the use of communication match well with our requirements 1 (the need to control for ex-ante and ex-post information about belief of others) and 3 (the use of verbal communication to make a social norm salient). Unfortunately, these features are not sufficient to disentangle the role of the desire for esteem from the intrinsic desire to meet others' expectations since both types of subjects might choose the same actions (in contrast with our requirement 2)^10^.

In order to meet also our requirement 2, we have modified the original C&D design in two ways.

First, we have made *the action chosen by B observable*. In this version of the risky Trust game with “exposure,” A was informed at the end of the game about the action that B has chosen (see the instructions in the Supplementary Materials for further details). Tadelis (2011) and Bracht and Regner (2013) have contrasted the risky Trust game with and without exposure since the former, but not the latter, allows exploring a concern for ex-post perception, i.e., a concern for what the others *think* of oneself at the end of the interaction. As clarified above, such a concern should be especially appealing to those who care for others' esteem because being perceived as a “bad” player—one who has decided not to roll—would entail a withdrawal of esteem, and would elicit shame.

**Figure 2:**
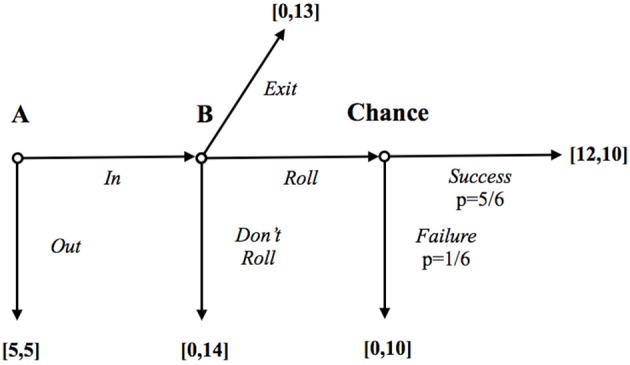
**Trust game with exposure and a costly exit option**.

In addition to exposure, moreover, we have also added the possibility for B players to *misinform* their matched A about their actual choice. In particular, in our design, *each B subject had the option to deceive the matched A subject*. In particular, each B could decide to pay a cost for letting the matched A believe that a bad outcome was due to an unlucky dice roll and not to B's choice to keep the whole pot for himself. Since only B players were informed of this *exit* option, the resulting game was one of incomplete information in which the structure of the social interaction was not common knowledge (see Figure 2).

As with exposure, the exit option has been introduced to manipulate A's ex-post information, and should thus be appealing to players who are motivated by others' esteem but *not to those who mainly care for not disappointing others' expectations per se*. Players mainly concerned with esteem could thus exploit the “wiggle room” created by the exit option (Dana et al., 2006; for a similar motivation but different design see also Bicchieri and Chavez, 2010). As a consequence, in our design with the costly exit option, B players who chose the cooperative outcome (i.e., ROLL option) *were driven by the desire to meet others' expectations*^11^.

Finally, since, as we have suggested above, this desire can be understood in two different ways—as guilt aversion or as perceived legitimacy—we have also measured both empirical and normative expectations of participants. By identifying which expectation was in fact related to actual behavior, measuring expectations has been crucial to determine the motivation that is more relevant for different types of subjects (see below, for further details).

### Main treatments

In order to study the role of different motivations for social norm compliance, we have designed three separate treatments: *Message* & *Exit, Message* and *Exit*. In addition to these treatments we have also replicated C&D's original design that is identical to our *Message* treatment but without exposure (*Message C*&*D* treatment). This replication has been conducted to check our assumption that, notwithstanding its theoretical soundness, C&D design cannot disentangle the desire for esteem from the desire to meet others' expectation. As a consequence, the replication has provided an indirect validation of our new design.

In the *Message* & *Exit* treatment we have employed our trust game with communication, exposure and the exit option. In particular, B subjects had the option to let As believe that their outcome (0) has been the result of an unlucky dice roll instead of the consequence of B's deterministic choice not to roll. The exit option had the following payoffs for A and B subjects: 0€ and 13€. Thus the exit option was costly: by choosing EXIT, B obtained a lower material payoff (13€) than by choosing DON'T ROLL (14€).

The *Exit* treatment was identical to *Message* & *Exit*, with the only difference that the no communication was allowed.

**Figure 3:**
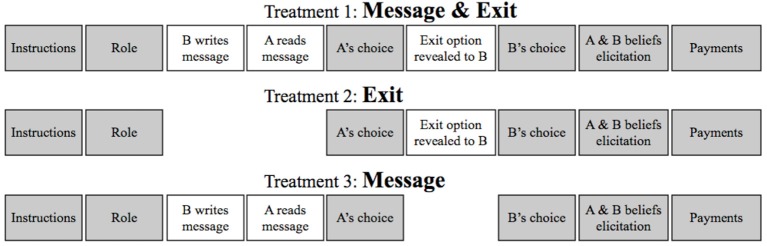
**Timeline of the three main treatments used in the experiment**. The additional *Message (C*&*D*) treatment has all the same features of *Message* but without exposure (i.e., A is not informed about B's actual choice).

In the *Message* treatment, we have employed the risky trust game with exposure and communication but without the exit option. Figure 3 summarizes the timeline of the three main treatments and highlights the relevant manipulations.

As it can be verified in Figure 3, the existence of the exit option is revealed to B subjects only in two treatments, and only after all Bs had decided whether or not to send a message (in *Message* & *Exit*) and after As had decided whether to choose IN or OUT (*Exit* and *Message* & *Exit*). Thus, the existence of this option cannot influence either As' choices or Bs' decisions of whether to send a message and, if so, which one.

### Belief elicitation procedure

To determine whether the desire to meet others' expectations depends on others' empirical expectations (guilt aversion) or normative ones (perceived legitimacy), we have measured: (1) *A's empirical expectations on B* and *B's second-order empirical expectations* (i.e., what B believes that A expects that B will do); (2) *A's personal normative beliefs* (i.e., what each A privately thinks a B ought to do) and *B's second-order normative expectations* (i.e., what B believes that A thinks that B ought to do). Moreover, given that we are interested in social norm compliance, (3) we have also elicited *the empirical and normative expectations between B players*, i.e., between trustees.

Following C&D procedure, expectations were measured as follows. After collecting As' and Bs' strategic choices, participants were invited to make guesses about the choices of their counterparts and their predictions were incentivized. A subjects were asked to guess the proportion of B subjects who will choose to ROLL (As' first order empirical expectations on Bs); while B subjects were asked to guess the average guess made by As who had chosen IN (Bs' second-order empirical expectations on As). We used the same procedure also to elicit and measure normative expectations. A subjects were asked if they felt *entitled* that B chose ROLL (A's personal normative beliefs); while Bs were asked to guess the percentage of As who felt entitled that Bs chose to ROLL (Bs' second-order normative expectations on As). Finally also the normative beliefs of B subjects were elicited, i.e., B's belief that he or she ought to ROLL, and the second-order normative expectations between Bs, i.e., a B subject's belief about other Bs' beliefs that a B ought to ROLL. With the exception of As' or Bs' personal normative beliefs, all other beliefs have been elicited with an incentive compatible procedure: players received additional 5€ only if their guess differed no more than 5% points from the actual percentage.

By measuring these beliefs between treatments with and without communication, we could thus verify whether our assumption that communication makes a social norm salient was confirmed. More importantly, we could also observe which kind of expectations was in fact related to actual behavior.

Table 1 summarizes the belief elicitation task.

**Table 1:** **Questions used to elicit different kinds of expectations**.

**Question**	**Expectation**
**A SUBJECTS**	
Guess the % of B's who chose Roll	A's first-order empirical expectation on B's behavior
Do you feel entitled that B chose Roll?	A's personal normative belief
**B SUBJECTS**	
Guess the % of Bs who choose Roll indicated by As	B's second-order empirical expectations (belief about A's belief)
Guess the % of Bs who chose Roll	B's first-order empirical expectation on other Bs
Do you think you ought to choose Roll?	B's personal normative belief
Guess the % of As who feel entitled that B chose Roll	B's second-order normative expectations on A (B's belief about A's personal normative belief)
Guess the % of Bs who think they ought to choose Roll	B's second-order normative expectation on other Bs (B's belief about other Bs' personal normative beliefs)

*Original questions were in Italian*.

### Coding scheme for messages

In all treatments with communication (*Message, Message* & *Exit*, and *Message C*&*D*), Bs' messages have been coded according to four categories: “Promise,” “Fairness,” “Mutual Advantage,” and “Irrelevant.” A message has been classified as a “Promise” if B *explicitly* stated his or her intention to ROLL if A had chosen IN. If no explicit reference to B's action in the future was made but the message contained a judgment about some normative feature of the outcome, it has been classified as “Fairness.” Finally, if B attempted to influence A by suggesting that the outcome induced by the IN-ROLL profile would have benefited both members of the dyad, it has been classified as an appeal to “Mutual Advantage”^12^. All other messages that did not fall in these three categories have been classified as “Irrelevant.” The coding has been realized by two independent judges, who were blind to the aims of the study. The coding scheme was decided before data collection and has been devised to check for social norms that are frequently considered relevant in the contexts of trust games.

### Participants and procedures

The experiments have been conducted at the CESARE lab of LUISS University in Rome, Italy. We ran 8 pen and paper sessions between January 2013 and October 2013 with a total amount of 318 subjects. All participants provided written informed consent. The study was carried out in accordance with the ethical guidelines of the Italian Association of Psychology (AIP) and approved by the Ethics Committee of ISTC-CNR.

Each session involved 40 subjects in total, with 20 A subjects and 20 B subjects (with the exception of one session of the *Message* treatment which had 38 subjects, with 19 A subjects and 19 B subjects). A subjects were in the same room and could see (and count) each other; the same was true for B subjects.

**Figure 4:**
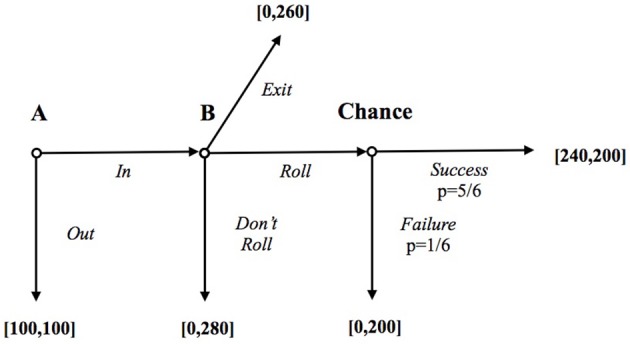
**The game with payoffs expressed in experimental tokens (conversion rate: 1 token = 0.05€)**.

Subjects were all Italian undergraduate students (65.9% from Economics), with 43.8% females. We employed a between-subjects design: no individual participated in more than one session. Payoffs were expressed in experimental tokens and each token was converted into 0.05€ (see Figure 4). In each session, the participants were paid a 2€ show-up fee, plus their earnings from the experiment. The average payment per participant was 8.41€ (plus the show-up fee) and the sessions averaged approximately 1 h.

At the beginning of each session, participants were welcomed in two separate rooms and, once all of them were seated, the instructions were handed to them in written form before being read aloud by two experimenters. All subjects completed a final questionnaire containing demographic information, personality details (i.e., measures of happiness, of generalized trust, of guilt proneness, and risk aversion) and self-reported motivation for the decisions made in the experiment.

In each session, participants were referred either as A subjects or as B subjects. A coin was tossed to determine which room was A and which was B. Participants were provided with identification numbers and were informed that these numbers would have been used to determine pairings (one A with one B) and to track decisions. Participants in the role of B made their choices without knowing A's actual choice of IN or OUT (strategy method), but they were told that Bs' choice would be immaterial if A had chosen OUT. To ensure anonymity, after all the decisions had been collected, a 6-sided dice was rolled for each B irrespective of his or her actual decision (i.e., for those B who chose DON'T ROLL or EXIT, rolling the dice was inconsequential).

## Main hypotheses

Given that our design is aimed at studying social norm compliance and at disentangling the role of the desire for others' esteem from that of the desire to meet others' expectations, it follows that:

*Hypothesis 1(a): the rate of ROLL choices is higher both in* Message *and in* Message & Exit *with respect to* Exit.

In other words, if communication makes a social norm salient, the treatments in which Bs can send a message to As will elicit more social norm compliance (i.e., more ROLL choices) than when no communication is possible and thus any relevant social norm would be more ambiguous.

*Hypothesis 1(b): the strength of As' and Bs' empirical and normative expectations will be higher both in* Message *and in* Message & Exit *with respect to* Exit.

This means that, whenever communication is possible and a social norm is more salient, both empirical and normative expectations will be inflated. Alternatively put, communication strengthens As' expectations that B will ROLL, B's beliefs about these expectations, A's beliefs to be entitled to B's rolling the dice, and B's beliefs about these normative expectations. The same holds also for B's expectations on other Bs.

*Hypothesis 2: “Promises” will be more predictive of ROLL choices than other kind of messages*.

Again, if communication can be used to make a social norm salient, especially in the context of a Trust game in which one aims to induce another person's reliance on oneself, B's assurances that he or she will indeed ROLL the dice if the other chooses IN (i.e., “promises”) will unambiguously invoke a norm of promise-keeping or of keeping one's word^13^. Thus, we expect that by choosing to send a message that can be interpreted as the intention to ROLL, the rate of social norm compliance (i.e., the decision to conform to the rule of keeping one's word and to choose in fact ROLL) will be higher.

*Hypothesis 3: the rate of ROLL choices is higher in* Message *than in* Message & Exit.

While in *Message* both the desire for others' esteem and the desire to meet others' expectations might motivate social norm compliance, in *Message* & *Exit* only those who are mainly motivated to meet others' expectations will choose to ROLL while those that are mainly motivated by others' esteem will choose EXIT. If this is true, Hypothesis 3 follows.

Taken together the confirmation of Hypotheses 1, 2 and 3 would validate our design and would offer evidence that we have been able to isolate subjects mainly driven by the desire for others' esteem from those mainly driven by the desire to meet others' expectations.

Finally, our design is also intended to empirically establish whether the desire to meet others' expectations depends on *empirical* expectations (as suggested by guilt aversion theory) or on *normative* ones (as suggested by perceived legitimacy). As a consequence:

*Hypothesis 4(a): If guilt aversion is true, ROLL choices in* Message & Exit *will correlate with B's beliefs about A's empirical expectations (B's second-order empirical expectations)*.

*Hypothesis 4(b): If perceived legitimacy is true, ROLL choices in* Message & Exit *will correlate with (1) B's beliefs about A's normative expectations and with (2) B's beliefs about normative expectations of other Bs*.

In other words, if the desire to meet others' expectations is a form of guilt aversion, social norm compliance (i.e., ROLL choices in *Message* & *Exit*) is explained by B's motivation not to disappoint A's payoff expectations (A's empirical expectations). In contrast, if the same desire is understood as perceived legitimacy, social norm compliance is explained by B's motivation not to disappoint A's *normative* expectations, that is, those expectations that B perceives as legitimate irrespective of A's payoff expectations. To put it differently, while guilt aversion predicts that Bs who choose ROLL in *Message* & *Exit* are disposed to comply with the social norm to avoid the psychological distress they would feel if A received less than expected (i.e., a form of altruism), perceived legitimacy predicts that those same Bs are disposed to comply with the social norm merely because they perceive As' normative expectations as legitimate.

## Results

### Result 1(A): communication increases trust and trustworthiness

Results show that A subjects, the Trustors chose IN with a frequency that equals 43.5% (17 of 39), whereas the percentage of Bs, the Trustees who decided to ROLL is 53.8% (21 of 39) in *Message* treatment; percentages are 22.5% (9 of 40) and 17.5% (7 of 40) in the *Exit* treatment, and 42.5% (17 of 40), and 32.5% (13 of 40) in the *Message* & *Exit* treatment, respectively. B subjects chose EXIT in 22.5% (9 out of 40) cases in the *Exit* treatment, and 20% (8 out of 40) in the *Message* & *Exit* Treatment.

**Figure 5:**
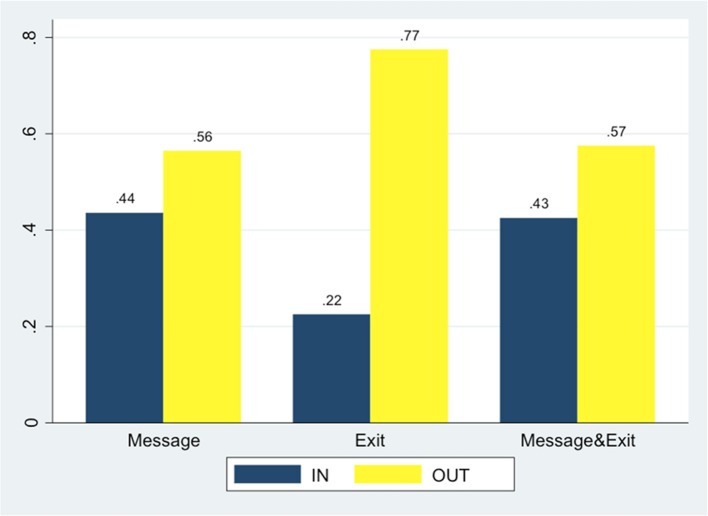
**As' choices in different treatments**.

Figure 5 summarizes A's choices in *Message, Exit, Message* & *Exit* treatments. Results of *Message (C*&*D*) will be discussed separately in the next section.

In *Exit*, where there is no opportunity to receive a message, A subjects chose IN significantly less than in *Message* and *Message* & *Exit*, where B subjects could send them a message (*z* one-sided test, *p* = 0.002, and *p* = 0.003 respectively).

**Figure 6:**
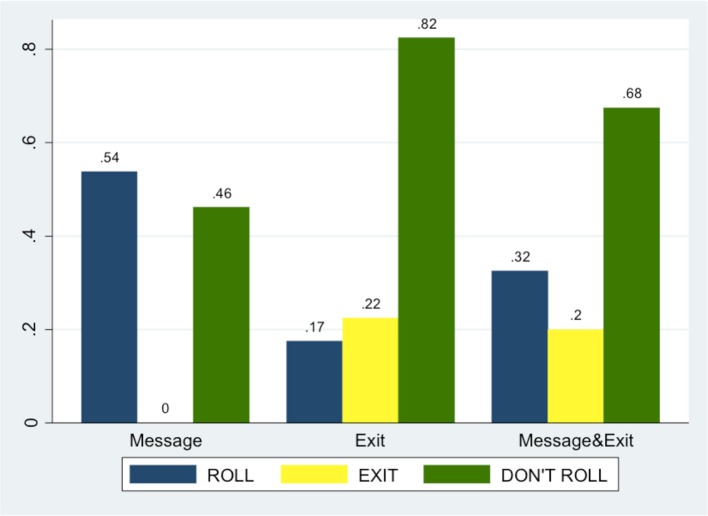
**Bs' choices in different treatments**.

Figure 6 summarizes Bs' choices in *Message, Exit, Message* & *Exit* treatments.

There is a significant difference in Bs' decisions to ROLL between *Exit* and *Message* (*p* = 0.000, *z* one-sided test), and—although at a lower degree of significance—between *Exit* and *Message* & *Exit* (*p* = 0.062, *z* one-sided test). For a comparison between *Message* and *Message* & *Exit*, see below the section on Result 3.

Thus, in line with previous research on the role of communication in social dilemma kind of situations (for reviews see Sally, 1995 and Balliet, 2010), these results confirm that communication significantly increases trust and trustworthiness, and hence also our Hypothesis 1(a).

### Result 1(B): communication strengthens empirical and normative expectations

**Figure 7:**
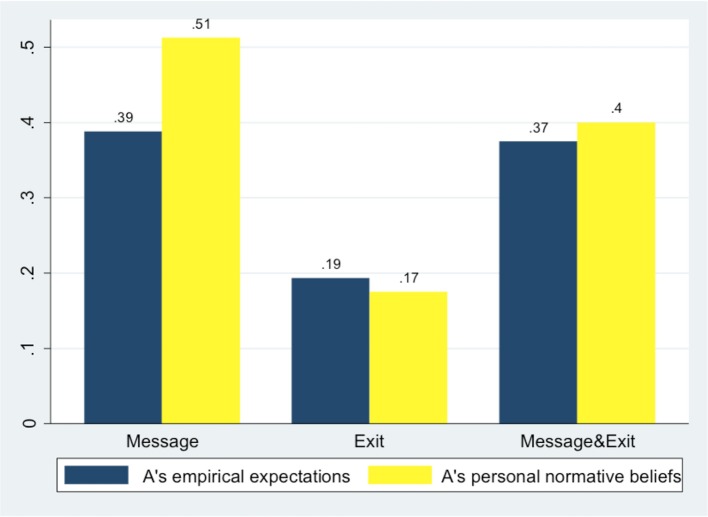
**A's empirical expectation on B's ROLL decisions and A's personal normative beliefs**.

Figure 7 compares A's empirical and normative expectations across treatments. See Table 1 above for a description of all the different beliefs and expectations elicited.

As' empirical expectations on Bs' behavior and their normative expectations of a trustworthy behavior have been inflated by the presence of the message: there is a significantly higher level of both beliefs when comparing *Message* & *Exit* with *Exit* (*p* = 0.000 and *p* = 0.027 respectively, *z* two-sided test) and *Message* with *Exit* (*p* = 0.000 and *p* = 0.002 respectively, *z* two-sided test). A's beliefs do not differ between *Message* & *Exit* and *Message*.

Furthermore, the level of As' empirical expectations and normative beliefs are generally not significantly different from each other across treatments, with the exception of *Message* where the difference is significant (*t* = −1.7193, *p* = 0.046, one-sample *t*-test). Since *Message* and *Message* & *Exit* differed only in the use of the exit option, but this option was ignored by As and was revealed to Bs only after their decision to send a message, the higher level of As' normative expectations is probably due to the kind of message that As received in this treatment. Indeed, in *Message*, B subjects promised more frequently than in *Message* & *Exit* (see **Figure 10** below).

**Figure 8:**
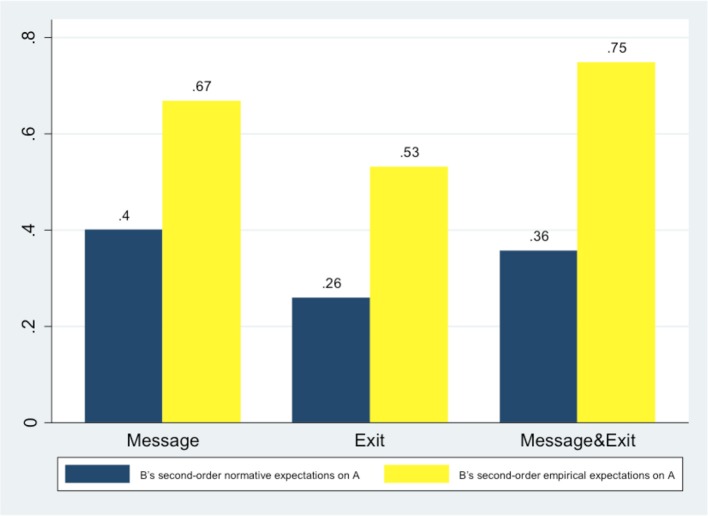
**Bs' normative expectations on As and second-order empirical expectations on As**.

**Figure 9:**
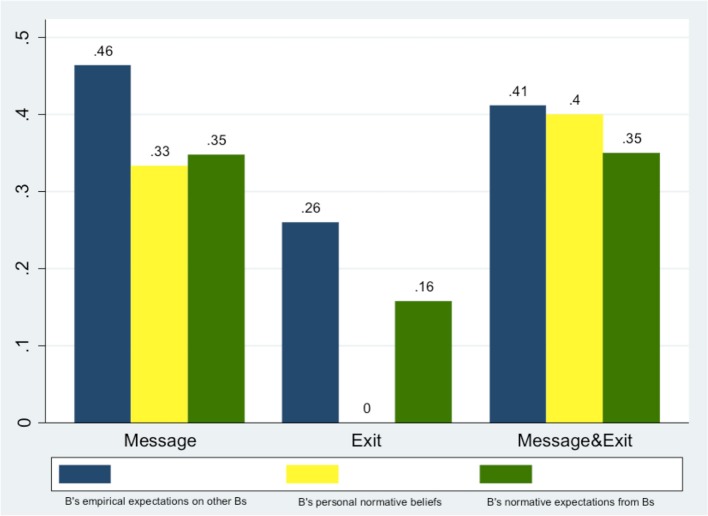
**Empirical and normative expectations among Bs about Bs' decisions to ROLL**.

Bs' second-order *normative* expectations and Bs' second-order *empirical* expectations on As are characterized by similar results, summarized in Figure 8. Figure 9 summarizes the average level of Bs empirical expectations on other Bs, their guesses about one another's expectations (second-order empirical expectations) and Bs' personal normative beliefs.

Overall these results confirm that communication has inflated both trustors' expectations (As' beliefs) and trustees' beliefs about trustors' beliefs (Bs' second-order empirical expectations on As). At the same time, communication has also boosted trustees' beliefs about trustors' normative expectations and the belief about what was normatively expected of them by other B subjects in the same role.

Importantly, we have also found that there is “consensus” (Bicchieri et al., 2011) between Bs' personal normative beliefs and B's second-order normative expectation on other Bs, but only in treatments where Bs could send a message: there is no significant effect in the *Exit* treatment, but there is a significant effect in *Message* & *Exit* (Spearman correlation test, with coef. = 0.304, *p* = 0.056) and in *Message* (Spearman correlation test, with coef. = 0.462, *p* = 0.003). Additionally, only within subjects in *Exit* there is a significant difference between Bs' personal normative beliefs and B's second-order normative expectation on other Bs (*t* = −4.888, *p* = 0.000, one-sample *t*-test), which confirms that without communication the social norm was less salient between subjects. These results support our Hypothesis 1(b) that communication makes a social norm salient by boosting the relevant expectations.

### Result 2: communication makes a social norm of promise keeping salient

In order to test for Hypothesis 2, we first explore how communication was actually employed. As far as the decision to send a message is concerned, there is no difference between *Message* and *Message* & *Exit*: 36 out of 39 write a message in *Message* & *Exit*, 35 out of 40 in *Message* (*p* = 0.240, *z* one-sided test).

**Figure 10:**
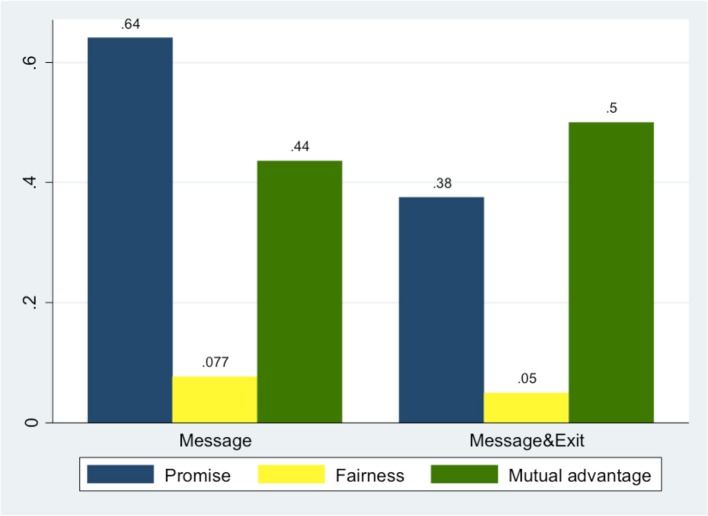
**Proportions of messages with different contents**.

With regard to the content of these messages, Figure 10 summarizes the frequency of each content category across treatments (for the coding scheme, see Section Coding Scheme for Messages).

B subjects in *Message* & *Exit* promised less frequently than in *Message* (43% vs. 70%, *p* = 0.009, *z* one-sided test); messages referring to fairness or mutual advantage were equally frequent in the messages of the two treatments (*p* = 0.312 and *p* = 0.285 respectively, *z* one-sided test).

Receiving a message did not increase the frequency of IN choices *per se* (*z* one-sided test, *p* = 0.306) but the probability to choose IN increased significantly when As received a message containing a promise (from 35.38 to 50% of IN choices, *p* = 0.046) or when B referred to a fair split of the pie (from 40.91 to 80% of IN choices, *p* = 0.043). There was no effect when the message appealed to mutual advantage in order to influence IN choices (*p* = 0.338).

More importantly, a message containing a promise has determined a significantly larger choice of ROLL (*z* one-sided test, *p* = 0.048). An appeal to outcome fairness or to mutual advantage did not affect either EXIT or ROLL choices of B subjects.

These results allow us to conclude that communication has made a social norm of promise-keeping especially salient (our Hypothesis 2), and that communication has influenced Bs' trustworthiness by motivating compliance primarily with this norm.

Moreover, considering that Bs were informed about the exit option only after having sent their messages, it is suggestive also that Bs who promised were less likely to choose EXIT: from 16.13 to 5% of EXIT choices (*p* = 0.0642, *z* one-sided test). A message referring to fairness or mutual advantage was not similarly effective (*p* = 0.220 and *p* = 0.176 respectively, *z* one-sided test). In other words, other possibly relevant norms like a norm of fairness or an appeal to mutual advantage do not seem to influence trustworthiness in this experiment.

### Result 3: social norm compliance can be driven both by the desire for others' esteem and by the desire to meet others' expectations

Following our Hypothesis 3 we have tested whether ROLL decisions significantly differ between *Message* and *Message* & *Exit* (see Figure 6). As expected, they do (*p* = 0.028, *z* one-sided test): B subjects chose to ROLL significantly more in *Message*.

More than 20% of Bs chose the EXIT option in both treatments where it was available (more precisely, 8 subjects out of 40 in *Message* & *Exit* and 9 out of 40 in *Exit*), and there is no difference in the use of this option between *Message* & *Exit* and *Exit* (*p* = 0.3940). Sending (or not) the message *per se* does not seem to affect the choice of the EXIT option.

Moreover, and more importantly for our aims, we find that there is no significant difference in DON'T ROLL choices across treatments (46.15% in *Message*, 60% in *Exit* and 47.5% in *Message* & *Exit*; *p* = 0.110, *p* = 0.452 and *p* = 0.132 respectively, *z* one-sided test).

Thus, given that, as we have shown before, subjects decided to ROLL significantly more when the exit option was not available, *we can infer that subjects who choose to EXIT belongs to the ROLL pool*: i.e., these are subjects that would have chosen to be trustworthy (i.e., to ROLL) if their violations were observable. This confirms our Hypothesis 3 and validates our design, whose aim is to disentangle players who comply with the social norm because of what others think of them—the desire for others' esteem—from players motivated not to disappoint others' expectations.

### Result 4: when nobody can monitor violations, compliance with a social norm is driven by the perceived legitimacy of normative expectations

Taken together Results 1, 2, and 3 allow us to conclude that our design has been successful in making a given social norm salient, in promoting social norm compliance, and in isolating two key motivations behind it. However, we still have to show whether the desire to meet others' expectations depends on others' *empirical* expectations (Hypothesis 4a) or *normative* ones (Hypothesis 4b).

Table 2 shows that, in general, there is a significant correlation between B's second-order *empirical* expectations on A only in case of *Message* treatment.

**Table 2:** **Correlation between Bs' second-order empirical expectations on A, Bs' empirical expectations on other Bs and Bs' behavior**.

	**Message**		**Exit**		**Message & Exit**	
	**On As**	**On other Bs**	**On As**	**On other Bs**	**On As**	**On other Bs**
**EXIT**						
Coefficient	–	–	0.054	0.058	0.085	0.215
(*p*-value)	–	–	(0.736)	(0.719)	(0.598)	(0.181)
**ROLL**						
Coefficient	0.487^***^	0.240	0.024	0.048	−0.105	0.147
(*p*-value)	(0.001)	(0.141)	(0.881)	(0.767)	(0.518)	(0.363)
**DON'T ROLL**						
Coefficient	−0.487^***^	−0.240	−0.065	−0.087	0.055	−0.267
(*p*-value)	(0.001)	(0.141)	(0.687)	(0.591)	(0.733)	(0.094)

*** *p < 0.01*.

Interestingly, if we pool together subjects who chose to ROLL and to EXIT (i.e., those who avoided to publicly violate the norm) in *Message* & *Exit*, the correlation between B's choice and B's second-order empirical expectation on A is significant as well (coef. 0.238, *p* = 0.035) like that with second-order empirical expectations on other Bs (coef. 0.248, *p* = 0.027). Since, as we have established before (see the previous section), the pool of subjects who chose to ROLL in *Message* includes also subjects that were motivated by others' esteem and were worried to lose it, we may conclude that the correlation between B's second-order empirical expectations and behavior cannot reliably be used as evidence for one motivation in particular. Moreover, if, in *Message*, we restrict the analysis to subjects who have sent a message containing a promise (i.e., those who should have mainly been moved by guilt aversion), the correlation between B's second-order empirical expectations on A and B's choice is not significant (coef. = 0.115, *p* = 0.582). On the other hand, both analyses suggest that our manipulation has been successful in disentangling the two key motivations.

In contrast, as Table 3 shows, Bs' choices are significantly correlated with Bs' second-order *normative* expectations both on As and Bs as well as with Bs' personal normative beliefs: Bs' willingness to comply with the social norm, and to ROLL, is increasing with their second-order normative expectations on As and on other Bs while their willingness to violate it, and choose DON'T ROLL, is increasing, the lower these normative expectations are.

**Table 3:** **Correlation between Bs' personal normative beliefs, Bs' perceived legitimacy of As' normative expectations and Bs' normative expectations on other Bs (B's belief that other Bs' believe that he ought to ROLL) and behavior**.

	**Message**			**Exit**			**Message & Exit**		
	**Personal normative belief**	**Normative expectations(on A)**	**Normative expectations (on other Bs)**	**Personal normative belief**	**Normative expectations(on A)**	**Normative expectations (on other Bs)**	**Personal normative belief**	**Normative expectations(on A)**	**Normative expectation (on other Bs)**
**EXIT**									
Coefficient	–	–	–	–	0.280^*^	−0.066	0.102	0.238	−0.219
(*p*-value)	–	–	–	(0.000)	(0.079)	(0.968)	(0.530)	(0.139)	(0.173)
**ROLL**									
Coefficient	0.218	0.204	0.232	–	0.141	0.014	0.287^*^	0.289^*^	0.331^**^
(*p*-value)	(0.182)	(0.210)	(0.154)	(0.000)	(0.382)	(0.930)	(0.072)	(0.070)	(0.036)
**DON'T ROLL**									
Coefficient	−0.218	−0.204	−0.232	–	−0.349^**^	−0.005	−0.367^**^	−0.349^**^	−0.265^*^
(*p*-value)	(0.182)	(0.210)	(0.154)	(0.000)	(0.027)	(0.972)	0.019	(0.027)	(0.097)

* p < 0.10;

** *p < 0.05 respectively*.

Taken together these results confirm Hypothesis 4(b) over the alternative 4(a), and thus indicate that the desire to meet others' expectations depends on *normative* expectations and not on *empirical* ones, i.e., on what others think one *ought* to do and not on what others think that one will *probably* do. In other words, perceived legitimacy explains social norm compliance, but guilt aversion does not.

### Can empirical expectations of others be perceived as illegitimate?

Given that guilt aversion theory predicts a positive correlation between second-order empirical expectations and behavior, B's willingness to ROLL should be increasing in his second-order empirical expectations: the stronger B's beliefs about A's expectations are, the more B should be moved to meet them. However, in our *Message* & *Exit* treatment Bs' second-order empirical expectations on As do not correlate with Bs' choice to ROLL. A possible reason that would explain the lack of such correlation is that As' empirical expectations might have been considered themselves ungrounded, and thus illegitimate, by Bs (for an analogous objection to guilt aversion see also Sugden, 2009, p. 270). In other words, if only an appropriate range of As' expectations are perceived by B subjects as justified, a correlation between second-order expectations and choices would be absent^14^. In order to test this additional hypothesis we have conducted the following analyses.

Considering all three treatments, on average, Bs who chose ROLL have higher second-order empirical expectations than Bs who chose DON'T ROLL: Bs who chose ROLL thought that 41% of As expected Bs to choose ROLL, Bs who chose DON'T ROLL thought that 29% of As expected Bs to choose ROLL, and Bs who chose EXIT thought that 33% of As expected Bs to choose ROLL. There is a significant difference between expectations of Bs who chose ROLL with respect to those who chose DON'T ROLL (*p* = 0.002, Mann-Whitney, two-tailed test for all the analyses in this section), but not between expectations of Bs who chose DON'T ROLL and those who chose EXIT (*p* = 0.793) or between Bs who chose ROLL and those who chose EXIT (*p* = 0.127)^15^.

Interestingly, however, if we build a sub-sample of Bs with high beliefs on As expectations (≤ 0.50) and another one of Bs with low expectations (>0.50), what emerges is that the second-order expectations of B subjects who chose ROLL (average expectation 30%) are significantly higher than those who chose DON'T ROLL (average expectation 19%) but only in the subsample in which these expectations are equal or below the 0.50 threshold (*p* = 0.003). In the sub-sample in which B subjects had high expectations (>0.50), there was no difference in the level of second-order empirical expectations between the subjects who chose ROLL (average expectation 68%) and DON'T ROLL (average expectation 69%), with *p* = 0.778^16^.

When considering the exit option, we observe exactly the opposite effect. B subjects who chose EXIT were those with the highest beliefs on As' empirical expectations: i.e., there is a significant difference in the level of second-order expectations between Bs who chose EXIT (average expectation 76%) and Bs who chose DON'T ROLL (average expectation 69%), and between Bs who choose EXIT and Bs who chose ROLL (average expectation 68%), but only when considering B subjects whose expectations on As were above the 0.50 threshold (*p* = 0.000)^17^.

Considering also that subjects who chose EXIT typically also did not promise, these results suggest that high expectations of A subjects, absent a relevant social norm, might have driven Bs to choose EXIT maybe because they perceived that too much was expected of them, i.e., such expectations were not perceived as reasonable.

### Replication of Charness and Dufwenberg (2006)

In addition to our main design (the trust game with exposure and costly exit option), we have also conducted an additional treatment to replicate the original Charness and Dufwenberg (2006)'s design: the *Message (C*&*D*) treatment. We used exactly the same procedure and instructions, with the following two exceptions:

- Earnings were expressed in tokens (then converted in euros when paying subjects at the end of the experiment). Nonetheless, the vector of payoffs respected exactly the same relative magnitude—across players and across outcomes—chosen in the *Message (5,5)* treatment by Charness and Dufwenberg (2006). Each token in our *Message (C*&*D*) treatment corresponded to 0.05 dollars in Charness and Dufwenberg (2006) *Message (5,5)* treatment (and were converted in exactly the same amount of euros when we paid subjects);
- Participants were divided in two separate rooms.

Results show that A subjects exhibited a frequency of IN choices that equals 65% (26 of 40), whereas the percentage of Bs who decided to ROLL is 42.5% (17 of 40). Messages were sent in the 87.5% (35 of 40) of cases: 57.14% (20 of 35) of them contained a promise.

When compared to our *Message* treatment, A subjects chose IN significantly more in *Message (C*&*D*): 43.6% vs. 65% (*z* one-sided test, *p* = 0.028), whereas there is no significant difference in Bs' choice to ROLL (*z* one-sided test, *p* = 0.158). Therefore, our experiment does not replicate the exposure effect reported by Tadelis (2011) and Bracht and Regner (2013).

More importantly for our aims, we have also not found any correlation between Bs' beliefs and their choices. Whereas, Charness and Dufwenberg (2006) found that Bs who chose ROLL made significantly higher guesses about As' guesses than did Bs who chose DON'T ROLL, in our experiment there is no significant correlation between Bs' second-order beliefs and Bs' choice to ROLL (coef. = 0.222, *p* = 0.168). Furthermore, also Bs' second-order normative expectations are not significantly correlated with their ROLL choices (coef. = 0.216, *p* = 0.180). Our results are thus in agreement with Ellingsen et al. (2010) and Kawagoe and Narita (2014) who fail to report any significant correlation between second-order expectations and choices in the original C&D design.

Since the experimental conditions of our *Message (C*&*D*) are identical (with the exception of having participants seated in two separate rooms), the difference in this result might be ascribed to higher protection of anonymity that separating As from Bs in two rooms allows^18^. Sitting in the same room and being able to look at each other might have caused Bs to doubt that their choices were really obscure to As at the end of the experiment in Charness and Dufwenberg (2006)'s setup; this is less likely to occur in our replication. We conclude that in our *Message* treatment the exposure condition is responsible for the correlation between second-order empirical expectations and choices, and that this correlation mainly reflects the importance of the desire for others' esteem rather than guilt aversion.

## Discussion

Relying on the role that verbal communication plays in making a norm salient (Bicchieri, 2002), our design has been able to disentangle (1) the role in social norm compliance of the desire for others' esteem from that played by the desire to meet others' expectations and (2) to test two alternative ways of understanding the latter motivation. Results indicate that both motivations can in fact support social norm compliance (Result 3), but that only the desire to meet others' expectation can induce compliance even when one could violate with no material or immaterial sanction in sight. Moreover, we have shown that such desire depends on the normative expectations that other people have on oneself (Result 4). Thus, it is the *perceived legitimacy* of such expectations to motivate compliance, and not—as guilt aversion theory suggests (Sugden, 2000; Charness and Dufwenberg, 2006)—an altruistic aversion to disappoint others.

As a consequence, the evidence collected in this study is not compatible with Charness and Dufwenberg's conjecture that “guilt aversion may provide a form of microfoundation” for social norm compliance (Charness and Dufwenberg, 2006, p. 1596), though it does not exclude that dynamic psychological game theory (Battigalli and Dufwenberg, 2009) could be the right tool to model such microfoundation. Bicchieri and Sontuoso (2015), for example, have proposed a model of “conditionally conformist preferences” in which a player, B, who is considering whether or not to violate a social norm, anticipate the disutility he would experience if he were to disappoint others' payoff expectations. Crucially, in this model, the payoff expectations of other players are formed on the assumption that B will follow the operative social norm, and thus B's *utility is a function of his second-order normative expectations*.

We would also like to emphasize that our results question the role of guilt aversion as a motivation for social norm compliance but are not necessarily in contrast with guilt aversion as a relevant motivation in other contexts. Here the crucial point is that the kind of guilt modeled by guilt aversion theory presupposes a form of caring for another person's fate that seems to be more common between friends than between anonymous strangers in one-shot encounters. Actually, psychologists of emotions distinguish between two kinds of guilt: *guilt from harm* and *guilt from norm violation* (Miceli and Castelfranchi, 1998; for a review see Carnì et al., 2013). Guilt aversion theory has been mainly motivated by the so-called “interpersonal perspective” of Baumeister et al. (1994), which posits that an important kind of guilt results from the awareness of having caused unjustified harm to another. This feeling is based on empathy and compassion (Weiss, 1986) and is predicted to be a function of the social distance between people. If so, guilt from harm is what guilt aversion theory aims to model.

A quite different origin of guilt feelings is due, however, to the mere violation of a norm. Interestingly, one can experience this kind of guilt *even when breaking the norm is not observable and does not harm anybody else*. Think, for instance, at the violation of dietary norms socially enforced in ethno-religious groups like, for instance, Jews. Who will be harmed, should one group member eat some pork in private? If nobody is watching, nobody will be harmed, nor can be offended. Still, those who contemplate the violation of this norm might anticipate the guilt they would experience were they to eat pork anyway. In this perspective, the perceived legitimacy of others' expectations on our behavior might indeed induce guilt if one were to disappoint them even in absence of whatever empathetic identification with a victim.

Interestingly, recent neuroscientific evidence suggests that guilt from harm and guilt from norm violation might even be processed differently in the brain (Basile et al., 2011), and that neuronal networks that are selectively activated when experiencing guilt from norm violation, like the left and right insula, are also activated in a task similar to the one we have used in this study (Chang et al., 2011).

## Conclusion

Even if it is often contrary to ones' own material self-interest, people recurrently comply with social norms in everyday interactions. Plausibly, people behave as socially expected for multiple reasons, though different individuals might be predominantly moved by different concerns when deciding whether to comply with a social norm.

Aside from fear of peer punishment, which—if not at all overestimated (Guala, 2012)—certainly is the most explored explanation, we have focused on two alternatives that have attracted less attention: the desire for others' esteem (and to avoid shame) and a desire to meet others' expectations on oneself. Theoretically, it has been shown that both motivations could be sufficient to sustain social norms (Bernheim, 1994; Sugden, 2004; Bicchieri, 2006), but their empirical relevance is not known. Focusing on these motivations is interesting also because each might help to explain compliance with social norms in different contexts. If a desire to meet others' expectations exists, for instance, it would explain why some people comply with social norms even if nobody is there to monitor. Though establishing the existence of such desire is important in itself, it is however unclear what exactly its scope is: does this desire depend on others' *empirical* or *normative* expectations on oneself?

Here we have gathered new evidence that the desire to meet others' expectations is a crucial motivation for social norm compliance and that the *perceived legitimacy* of others' normative expectations is what sustains it, at least when no one is around to police.

### Conflict of interest statement

The authors declare that the research was conducted in the absence of any commercial or financial relationships that could be construed as a potential conflict of interest.

## Footnotes

^1^An individual's status can be also be interpreted as his *reputation*, and it can be used by peers to administer punishment of violations. This is another mechanism that supports the emergence and stability of social norms. See Kandori (1992).

^2^That this cost is tied to the waiter's expectations and not to a more general altruistic attitude toward a worker's welfare is evident if one considers that no pressure is felt to tip a shop assistant who typically does not expect it.

^3^It is essential to Sugden's theory that this aversion does not presuppose any belief that one *ought* to do the socially expected action, but only that such action is what others have reason to believe that one *will* do. This is why he considers normative expectations as a special kind of empirical ones (i.e., plain predictions). For counterarguments, see Anderson (2000), Verbeek (2002), Tummolini et al. (2013), and Colombo (2014).

^4^These are second-order beliefs that should be distinguished from one's own personal normative belief: my belief that *others believe* that I ought to do *A* vs. my belief that I ought to do *A*.

^5^For the notion of norm acceptance see, for instance, Gibbard (1990).

^6^*For more insights on what perceived legitimacy amounts to, see the Discussion below*.

^7^For clarity, in what follows we will mention only “guilt aversion” to refer to the construal of the intrinsic desire to meet others' expectations offered in Sugden (2000, 2004) and in Charness and Dufwenberg (2006). The main difference between the two proposals is just one of focus: while Sugden models the motivation as an aversion to be the target of others' resentment, Charness and Dufwenberg emphasize the distress one would feel for others' disappointment. This distinction does not seem to have empirical consequences for the aims of this paper.

^8^Consistently, Dufwenberg et al. (2011) have found that framing influence behaviors by influencing beliefs first, and have suggested that verbal communication might indeed operate as kind of framing effect endogenously produced by the communicating parties.

^9^This structure is intended to represent a situation in which one person, A, is considering whether to form a partnership with another one, B, in order to realize a joint project. The success of the joint project is in part due to chance and in part to how much effort B will exert. Since B's effort is private information, B might decide spare it.

^10^Actually, since a trustor cannot know whether or not a trustee has decided to ROLL, in C&D design the trustor cannot know whether a given trustee has been in fact good or bad. Hence, those trustees who are mainly concerned with trustors' ex-post beliefs about them should be assured that they will not lose their status. However, we suggest that, in practice, it is very difficult to control for these inferences and that subtle decisions made by experimenters might have unexpected epistemic consequences. See below our replication of C&D design for relevant evidence.

^11^It should be noted that, like in Dana et al. (2006) and in Bicchieri and Chavez (2010), our design employed a small form of experimental deception since a participant, who was randomly assigned the A role, was not informed in the experimental instructions about the complete structure of the social situation he or she was going to face. The decision however to deceive one's partner by choosing the exit option was intentionally and autonomously made by subjects playing the B role.

^12^Sugden (2011) has recently suggested that people may also display a motivation of “mutual advantage” when interacting in situations in which there is an outcome that is induced if they act jointly and that benefits all participants relative to a benchmark outcome that each can obtain independently from others' actions. The Trust game has precisely this feature.

^13^See Tummolini et al. (2013) for an extensive discussion of the normative consequences of inducing reliance on oneself in these contexts. See also Scanlon (1990) and Thomson (1990) for the special case of promises.

^14^Another possible reason for the lack of correlation between second-order empirical expectations and behavior in this game is that there is also incomplete information about Bs' “guilt types”. Indeed, Attanasi et al. (2013) have shown that with incomplete information about guilt-types, B subjects should display more dispersed beliefs and more heterogeneity of behavior. Interestingly, they also show that it exists a parameter region “where B subjects would cooperate and hold high second-order beliefs under complete information but exhibit less cooperative behavior and intermediate second-order beliefs under incomplete information” (p. 23). For the formal analysis of a trust game with incomplete information about guilt-types see Attanasi et al. (2015). Since in our design we do not control for information about guilt-types, we cannot exclude this alternative interpretation of our results.

^15^The latter non-significance is likely due—at least partially—to the limited number of subjects who chose EXIT.

^16^In the subsample of Bs with low expectations (≤ 0.50), 29% of subjects chose ROLL, 49% of subjects chose DON'T ROLL and 13% of subjects chose EXIT.

^17^In the subsample of Bs with high expectations (>0.50), 43% of subjects chose ROLL, 43% of subjects chose DON'T ROLL and 14% of subjects chose EXIT.

^18^As suggested before, a different explanation for this replication failure points to the fact that these designs are all characterized by incomplete information about guilt-types of participants. For a design in which complete and incomplete information about guilt-types is controlled, see Attanasi et al. (2013).
