# Supplementary material for: Perceived legitimacy of normative expectations motivates compliance with social norms when nobody is watching
Source: Front Psychol. 2015 Oct 6;6:1413. doi: 10.3389/fpsyg.2015.01413 (PMC4593938; doi:10.3389/fpsyg.2015.01413)
Supplement: Supplementary file 1 [file DataSheet1.DOCX]

**Appendix: INSTRUCTIONS**

*These instructions were used for* Message *and* Message & Exit *treatments. We indicate differences with the instructions used in the* Exit *treatment in italics, whereas differences with the instructions of* Message (C&D) *are emphasized in bold.*

Thank you for participating to this experiment. The purpose of this experiment is to study how people make decisions in a particular situation. Feel free to ask us questions as they arise, by raising your hand. Please do not speak to other participants during the experiment. The decisions you will take (that will be described below) will allow you to earn tokens. Each token corresponds to 0.05 euros.

The experiment takes place in two separate rooms. During the session, you will be paired with another person who is located in the other room. However, no participant will ever know the identity of the person with whom he or she is paired. In each pair, one person will have the role of A, and the other will have the role of B. The amount of money you earn depends on the decisions made in your pair. The attribution of the role of A or B will occur at a certain point during the experimental session through the roll of a dice, whose outcome will determine in which room subjects in the role of A and B are located, respectively.

How many tokens will you earn?

In each pair, on the designated decision sheet, each person A will indicate whether he or she wishes to choose IN or OUT. If A chooses OUT, the game ends and A and B each receive 100 tokens. If A choses IN, the subjects B in the same pair can choose between ROLL and DON’T ROLL. If A has chosen IN and B chooses DON’T ROLL, then B receives 280 tokens and A receives 0 tokens. If B chooses ROLL, B receives 200 tokens and rolls a six-sided dice to determine A’s payoff. If the dice comes up 1, A receives 0 tokens; if the dice comes up 2–6, A receives 240 tokens. This information is summarized in the chart below:

A gets B gets

A chooses OUT 100 100

A chooses IN, B chooses DON’T ROLL 0 280

A chooses IN, B chooses ROLL, dice=1 0 200

A chooses IN, B chooses ROLL, dice =2,3,4,5,6 240 200

Note that B will not know whether A has chosen IN or OUT before choosing between ROLL and DON’T ROLL; however, since B’s decision will only make a difference when A has chosen IN, we ask B’s to presume (for the purpose of making this decision) that A has chosen IN.

*Only for* Message *and* Message & Exit*:*

*[After the attribution of roles A and B:*

*Subjects B have the option to send a message to the subject A in their pair. Each B receives a blank sheet, on which a message can be written, if desired. Please print clearly if you wish to send a message to A. If you wish not to send a message, simply circle the letter B at the top of the sheet.*

*In these messages, no one is allowed to identify him or herself by name or number or gender or appearance. Subjects B have to indicate clearly the number that identifies their pair, otherwise the experimenter will not be able to give the message to its recipient. (The experimenter will monitor the messages. Violations - experimenter discretion - will result in B receiving 0 tokens). Other than these restrictions, B may say anything that he or she wishes in this message.]*

At this point, both A subjects and B subjects will be asked to make their own choice between the options allowed according to their role (IN or OUT for subjects A, ROLL or DON’T ROLL for subjects B) and following the procedure described above. **At the end of the game, A subjects will know what B subjects have chosen, and B subjects will know what subjects A have chosen.** **[In all treatments but *Message (C&D)*]**

Finally, both A subjects and B subjects will be asked to make predictions on the choices of other participants.

In all the sheets you receive, please remember to insert the number that identifies your pair and that has been assigned to you at the beginning of the experiment!

**[After that B subjects had decided whether or not to send their message to A subjects - in *Message & Exit* - and after that A subjects had chosen whether to play IN or OUT – in *Message & Exit* and in *Exit* -, B subjects were orally informed of the existence of the EXIT option and received an additional sheet of paper in which payoffs of the different choices were summarized and where B subjects could make their choices. B subjects were publicly informed that if a B subject chose the EXIT option, the matched A subject would have been informed that B had chosen to ROLL with 1 as the outcome of the dice. As a consequence of this choice, B subject would have earned 260 tokens and A subject would have received 0 tokens.]**
